# Clinical implications of the oncometabolite succinate in *SDHx*‐mutation carriers

**DOI:** 10.1111/cge.13553

**Published:** 2019-05-06

**Authors:** Karin Eijkelenkamp, Thamara E. Osinga, Thera P. Links, Anouk N.A. van der Horst‐Schrivers

**Affiliations:** ^1^ Department of Endocrinology and Metabolic Diseases University Medical Center Groningen, University of Groningen Groningen the Netherlands

**Keywords:** oncometabolites, paraganglioma, pheochromocytoma, SDH mutation, succinate

## Abstract

Succinate dehydrogenase (SDH) mutations lead to the accumulation of succinate, which acts as an oncometabolite. Germline *SDHx* mutations predispose to paraganglioma (PGL) and pheochromocytoma (PCC), as well as to renal cell carcinoma and gastro‐intestinal stromal tumors. The *SDHx* genes were the first tumor suppressor genes discovered which encode for a mitochondrial enzyme, thereby supporting Otto Warburg's hypothesis in 1926 that a direct link existed between mitochondrial dysfunction and cancer. Accumulation of succinate is the hallmark of tumorigenesis in PGL and PCC. Succinate accumulation inhibits several α‐ketoglutarate dioxygenases, thereby inducing the pseudohypoxia pathway and causing epigenetic changes. Moreover, SDH loss as a consequence of *SDHx* mutations can lead to reprogramming of cell metabolism. Metabolomics can be used as a diagnostic tool, as succinate and other metabolites can be measured in tumor tissue, plasma and urine with different techniques. Furthermore, these pathophysiological characteristics provide insight into therapeutic targets for metastatic disease. This review provides an overview of the pathophysiology and clinical implications of oncometabolite succinate in *SDHx* mutations.

## Graphical abstract

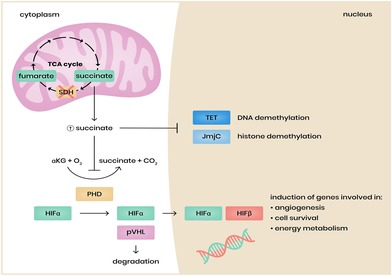

## 1 INTRODUCTION

Mutations of genes encoding for the succinate dehydrogenase (SDH) complex, associated with familial paraganglioma (PGL) and pheochromocytoma (PCC), lead to accumulation of succinate, which disturbs the metabolic regulation of the cell. Nowadays metabolic dysregulation is recognized as one of the eight hallmarks of cancer.^1^

Although germline mutations in *SDHx* genes are predominantly linked to PGL and PCC, these mutations also predispose to renal cell carcinoma (RCC), gastrointestinal stromal tumors (GISTs) and, possibly, pituitary adenomas. PCC, PGL and head and neck PGL (HNPGL) are rare neuroendocrine tumors arising from chromaffin cells that can synthesize and release catecholamines. Sympathetic PGLs are derived from sympathetic paraganglia in the chest, abdomen or pelvis. PCC are PGLs located in the adrenal medulla.^2^ HNPGLs are derived from parasympathetic paraganglia. Common locations for HNPGLs include the carotid body and the middle ear, as well as the vagus nerve and internal jugular vein. While parasympathetic PGLs are most often non‐functional tumors, PCC and sympathetic PGL release catecholamines into the circulation and can lead to severe (lethal) cardiovascular and cerebrovascular complications. Approximately, 40% of these tumors carry a germline mutation in one of more than 20 susceptibility genes, of which the *SDHx* genes are the most prevalent.^3^

In terms of genomic features, tumors related to *SDHx* mutations are classified as cluster I, along with Von Hippel Lindau (*VHL*), fumarate hydratase (*FH*), malate dehydrogenase 2 (*MDH2*), hypoxia induced factor (*HIF2α*) and isocitrate dehydrogenase (*IDH*)‐mutations and the recently identified *SLC25A11*.^4^ Cluster I germline mutations predispose to tumors characterized by a pseudohypoxic signature, in contrast to cluster II germline mutations, which are associated with abnormal kinase signaling pathways and include mutations in the genes of rearranged during transfection (*RET*), neurofibromatosis (*NF1*), transmembrane protein 127 (*TMEM127*), kinesin family member 1B (*KIF1B*), and MYC‐associated factor X (*MAX*). Cluster III is associated with the Wnt‐signaling pathway; it includes somatic mutations of cold shock domain‐containing E1 (*CSDE1*) and mastermind‐like transcriptional coactivator 3 (*MAML3*) fusion genes.^5^, ^6^

*SDHx* genes were the first to be recognized as tumor suppressor genes encoding a mitochondrial enzyme. This resulted in an upsurge of interest in the concept of aerobic glycolysis or the “Warburg effect,” reported by Otto Warburg in 1926, which is characterized by high glucose consumption and lactate production of cancer cells, even in the presence of oxygen.^7^ This metabolic dysregulation is in fact recognized as one of the eight hallmarks of cancer.

Defective SDH function triggers the accumulation of succinate, an intermediate metabolite of the tricarboxylic acid (TCA) cycle, which plays a crucial role in the generation of adenosine triphosphate (ATP) in mitochondria. Accumulation of succinate, along with other intermediate metabolites of the TCA cycle, can give rise to the development and progression of cancer. FH mutations lead to the accumulation of fumarate, and IDH mutations result in an accumulation of (R)‐2‐hydroxyglutarate. These oncometabolites modulate the activity of α‐ketoglutarate‐dependent dioxygenases, which are involved in the induction of the pseudohypoxia pathway and inhibit histones and DNA demethylases, resulting in a hypermethylator phenotype (also known as CpG island methylator phenotype [CIMP]). The *SLC25A11* gene encodes for a mitochondrial carrier protein that is part of the malate‐asparate shuttle (this shuttle regenerates NADH to allow complex I to function), mediating the transport of α‐ketoglutarate from the mitochondrial matrix to the cytoplasm in exchange with malate. Preliminary results show that in *SLC25A11*‐mutated cells aspartate and glutamate concentration is increased inducing the pseudohypoxic pathway and hypermethylation.^4^

Recognition of these pathophysiological characteristics provides unique opportunities for diagnostic and therapeutic strategies. Over the past years, several excellent reviews, such as those by Kucklova et al, Morin et al and Vicha et al, have discussed the pathophysiology of *SDHx*‐related tumors.^8^, ^9^, ^10^ In the current review, we first present a short summary of the SDH protein and the clinical features of *SDHx*‐mutation carriers. We then focus on the oncometabolite succinate and its pivotal role in tumorigenesis in *SDHx*‐related tumors, as well as on the implications for clinical practice, especially diagnostics and therapeutic options related to metastatic disease.

## 2 SUCCINATE DEHYDROGENASE

**Figure 1:**
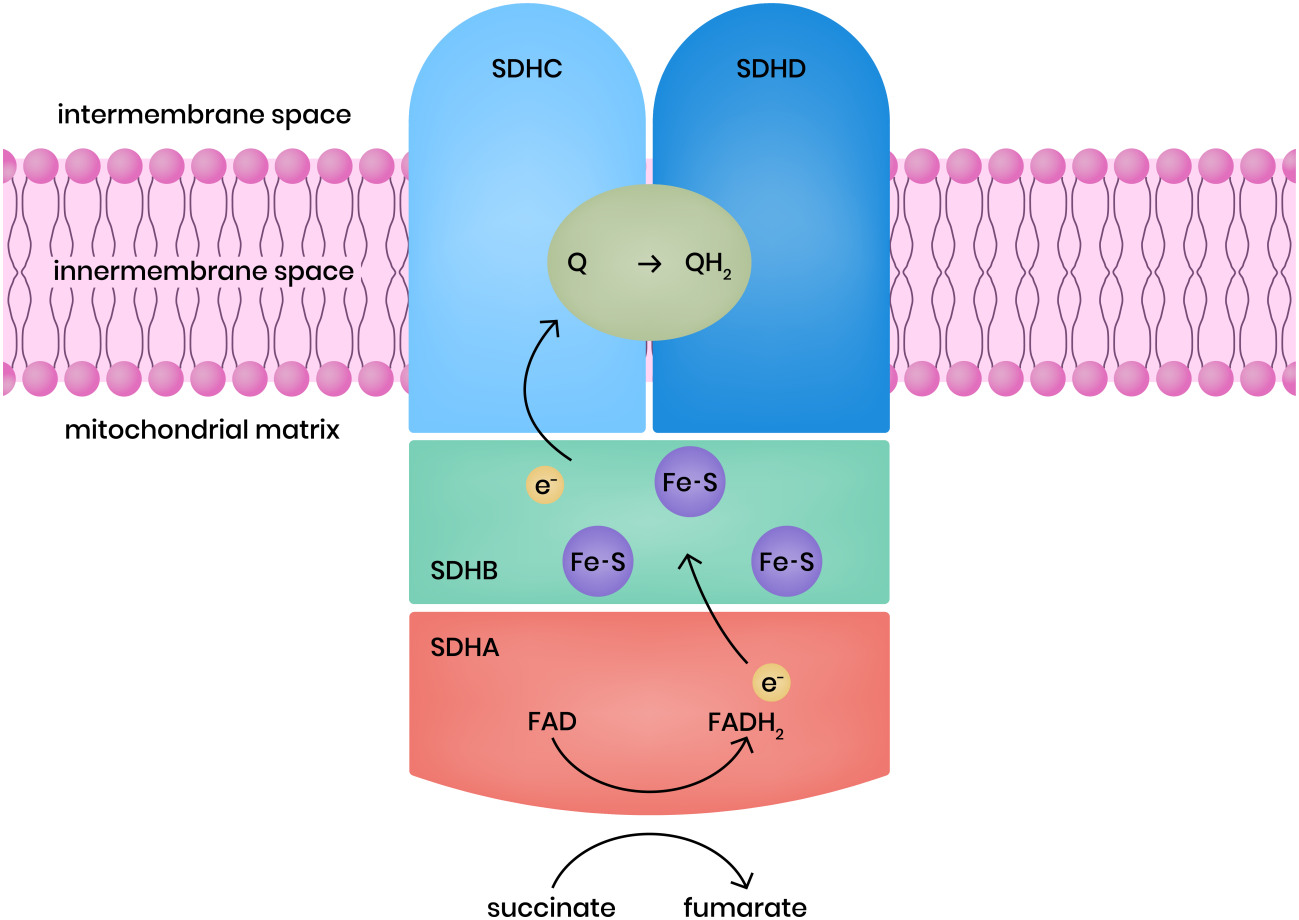
Succinate dehydrogenase (SDH) complex (simplified). The catalytic subunits SDH subunit A contains the flavin cofactor (FAD) which accepts electrons from succinate and passes them to Fe‐S center in the SDH subunit B subunit. The electrons are then passed the ubiquinone pool embedded in SDHC and SDHD subunits. Reduced Q (QH2 = ubiquinol) transfers electrons within the mitochondrial inner membrane space to complex III [Colour figure can be viewed at http://wileyonlinelibrary.com]

SDH is a hetero‐tetrameric mitochondrial enzyme that plays a role in the TCA cycle and in the mitochondrial electron transport chain as complex II (Figure 1). SDH catalyzes the oxidation of succinate to fumarate in the TCA cycle and transfers electrons to the ubiquinone (coenzyme Q) pool in the respiratory chain. SDH subunit A (SDHA) and subunit B (SDHB) comprise the catalytic subunits in the hydrophilic head that protrudes into the mitochondrial matrix. SDH subunit C (SDHC) and subunit D (SDHD) are the ubiquinone‐binding and membrane‐anchoring subunits. SDH assembly factor (SDHAF) is required for the flavination of SDHA, which is essential for the formation of the SDH complex. The *SDHA* gene is located on chromosome 5p15.33 and contains 16 exons.^11^ SDHA is the major catalytic subunit, converting succinate to fumarate. It contains the binding site for succinate. The gene encoding for *SDHB* is located on chromosome 1p35‐36.1 and has eight exons^12^; the SDHB protein contains three Fe‐S centers and mediates electron transfer to the ubiquinone pool. The gene encoding *SDHC* is located at 1q21 and has six exons,^13^ and the *SDHD* gene is located on chromosome 11q23 and has four exons.^14^ SDHC and SDHD bind ubiquinone, generating protons eventually leading to the production of ATP.

## 3 PHENOTYPE OF *SDHX* MUTATION CARRIERS

Although different *SDHx* mutations occur in genes encoding for a single enzyme, the clinical picture for each subunit differs with regard to penetrance, manifestations and rate of malignancy. International guidelines advice to screen all germline mutation carriers, however with different screenings strategies for different *SDHx* mutation carriers.^15^ Screenings advices do not only differ between the different mutations, but also over time, because studies on penetrance differ over time regarding the population studied (index included or not), the imaging methods used and the duration of follow‐up. Adherence to screening, leads to the detection of smaller PCC/PGL and might even lead to an improved survival for patients who develop metastases, although this is based on only few patients.^16^

Until now, a clear explanation for the difference of the clinical picture between different *SDHx* mutations is lacking, except for the hypothesis that the extent of SDH deficiency or loss depends on the subunit. Apart from the differences, all *SDHx* mutations are characterized by the (potential) presence of PGL/PCC. *SDHx*‐associated tumors harbor germline and somatic mutations, consistent with Knudson's second‐hit hypothesis.^12^ This hypothesis states that the combination of an inactivating germline mutation as a first hit, and somatic loss of function of the wild type allele as a second hit, is essential for tumor development.^17^ This second hit usually is an inactivation of the normal allele, that is, loss of 1p as was shown in a large genomic study.^18^

Germline *SDHx* mutations have been associated with neoplasms other than PGL/ PCC, such as RCC,^19^, ^20^, ^21^ GISTs and possibly pituitary adenoma.^22^, ^23^, ^24^ In addition, somatic *SDHx* mutations have been described in T‐cell leukemia.^25^ Because the discovery of *SDHx* genes is relatively recent, the full clinical phenotype of these carriers remains to be sufficiently clarified. The following paragraphs describe the currently known phenotype of each *SDHx* subunit (Table 1). The question of why *SDHx* mutations predispose to tumors in a select subset of tissues remains elusive.

**Table 1:** Phenotypic features of *SDHx* mutation carriers

	Prevalence (%)	Penetrance	Mode of inheritance	PCC	sPGL	HNPGL	Multifocality	Metastasis	Other tumors
SDHA	1–7	Low	AD	+	+	++	Rare	Yes	GIST, PA, NB
SDHB	8–10	Medium	AD	+	++	+	Rare	Frequent	GIST, RCC, PA
SDHC	1–2	Low	AD	+	+	++	Frequent	Rare	GIST, RCC, PA
SDHD	5–9	High	Paternal transmission^a^	+	+	++	Frequent	Rare	GIST, PA, RCC
SDHAF2	<1	Unknown	Paternal transmission^a^	−	−	++	Frequent	Unknown	PA

Abbreviations: AD, autosomal dominant; GIST, gastrointestinal stromal tumor; HNPGL, head and neck paraganglioma; NB, neuroblastoma; PA, pituitary adenoma; PCC, pheochromocytoma; RCC, renal cell carcinoma; SDH, succinate dehydrogenase; SDHAF2, SDH assembly factor; SDHA, SDH subunit A; SDHB, SDH subunit B; SDHC, SDH subunit C; SDHD, SDH subunit D; sPGL, sympathetic paraganglioma; −, manifestation (to our knowledge) not described in these mutation carriers.+, manifestation present in these mutation carriers; ++, most common manifestation of these mutation carriers.

a SDHD and SDHAF2 autosomal dominant with maternal imprinting.

### 3.1 SDHA mutations

Mutations in the *SDHA* gene were originally described as a cause of autosomal recessive juvenile encephalopathy, also known as Leigh syndrome.^26^ Later on, in 2010, a 32 year old woman with an abdominal PGL was reported to have a heterozygous *SDHA* germline mutation.^27^ Mutations in the *SDHA* gene remain a rare cause of PGL and account for 1% to 7% of all PGL cases.^28^, ^29^ About half of *SDHA* mutation carriers present with HNPGL, although sympathetic PGL and PCC are also reported.^30^, ^31^, ^32^ Recently, van der Tuin et al calculated the penetrance of *SDHA* mutation in a cohort comprising 86 patients (30 index and 56 non‐index patients). The penetrance for all manifestations was only 10% at age 70 in non‐index patients, but 50% at age 70 when both index and non‐index patients were included in the analysis.^32^

Metastatic disease was reported in 0% to 33% of PGL patients with *SDHA* mutations, although these reports included few patients (n = 4‐34).^31^, ^32^, ^33^, ^34^, ^35^, ^36^ GISTs and pituitary adenomas were reported in a small subset of patients.^24^, ^31^, ^32^, ^37^, ^38^ In a large pediatric GIST study of Boikos et al, a *SDHA* mutation, germline or somatic, was the most common molecular subtype^39^ Neuroblastoma was reported in one *SDHA* mutation carrier where it was possible to confirm loss of heterozygosity (LoH) in tumor tissue.^38^

### 3.2 SDHB mutations

Mutations in the *SDHB* gene are those most frequently found in PGL and account for about 10% of all cases of PGL.^28^ Most common manifestations are sympathetic PGLs (50%), whereas PCC and HNPGL occur less often (20%‐25% and 20%‐30%, respectively).^15^ Bilateral PCCs appear to be rare in *SDHB* mutation carriers. Penetrance of different manifestations decreases over time as more asymptomatic carriers are identified. Earlier studies of penetrance included mostly index patients, thereby overestimating the penetrance. A recent study by Andrews et al calculated the cumulative tumor risk in a large cohort of 673 *SDHB*‐ mutation carriers and corrected for such ascertainment bias by calculating not only the penetrance for only index patients, but also for a combination of index and non‐index patients.^40^ Their Kaplan‐Meier analysis showed an estimated risk for the combined manifestation of PCC, sympathetic PGL and HNPGL in non‐index patients to be 22% at age 60.^40^ In their retrospective cohort analysis (index and non‐index patients) the penetrance was 24% at age 60 years.^40^ Males seem to be slightly more at risk than females of developing a PGL.^40^, ^41^

*SDHB‐*related PGL/PCC are associated with a high risk of metastasis and poor prognosis. Earlier studies report a higher metastatic rate (31%‐97%)^42^, ^43^, ^44^, ^45^ than more recent studies.^40^ In a meta‐analysis including 12 studies comprising both asymptomatic *SDHB* carriers and carriers with manifest non‐metastatic disease, van Hulsteijn et al reported a metastatic rate of 17%.^46^ The risk of metastasis in HNPGL in *SDHB* mutation carriers appears to be lower compared to PGL developing at other sites.^15^ In a recent meta‐analysis of the outcomes of metastatic PGL and PCC, Hamidi et al found that the overall mortality in *SDHB* mutation carriers ranged from 35% to 55% (n = 96) compared to an overall mortality of 53% (95% confidence interval 43%‐63%) in the whole group of PGL/PCC.^47^ In the past, several studies have shown an association between *SDHB*‐related metastatic PGL/PCC and a shorter survival in patients compared to sporadic metastatic PGL/PCC.^48^, ^49^ In a recent analysis of Hescot et al, not the *SDHB* mutation status but hypersecretion of metanephrines and chromogranin A was found to be a significant prognostic factor for worst overall survival.^50^

Other *SDHB*‐associated tumors include RCC, although the risk for this manifestation seems low, varying between 4.7% and 8%.^21^, ^40^ GISTs are reported to occur in approximately 2% of *SDHB* carriers.^51^ Pituitary adenoma have been reported in nine cases, but only three had proven LoH (loss of heterozygosity) and abnormal SDHB expression, thus confirming involvement of SDHB mutation.^24^ Tufton et al reported a case of a *SDHB* mutation carrier with pituitary carcinoma.^52^

### 3.3 SDHC mutations

Mutations in the *SDHC* gene account for 1% to 2% of PGL/PCC cases.^28^ *SDHC* typically manifest as benign, non‐functional HNPGL, although sympathetic PGL and PCC are also reported.^53^, ^54^ Multiple HNPGL are common.^54^ Penetrance for all PGL/PCC manifestations in a cohort of 43 non‐index *SDHC* carriers was 25% at age 60.^40^

Although metastatic disease seems to be rare in *SDHC* mutation carriers, it has in a few cases been reported.^40^, ^55^, ^56^, ^57^ Eight RCC and multiple GISTs have been reported in *SDHC* carriers.^19^, ^58^, ^59^ Two cases of pituitary adenoma have been described, although for LoH studies no tissue was available to prove pathogenicity.^24^

### 3.4 SDHD mutations

A mutation in the *SDHD* gene accounts for approximately 5% to 9% of all cases of PGL/PCC.^28^, ^29^ This gene follows an autosomal dominant inheritance, modified by maternal imprinting. The predominant clinical feature of *SDHD* carriers is the development of (multiple) HNPGLs, as 85% of carriers develop tumors at this site.^51^ PCC and sympathetic PGL occur less frequently in 10% to 25% and 20% to 25% of carriers, respectively. Penetrance for 160 non‐index *SDHD* mutation carriers was 43% at age 60.^40^

Metastatic risk in *SDHD* carriers is low and occurs in 7% to 8% of cases.^15^, ^60^ Other associated tumors include RCC and GIST, although the lifetime risk for this manifestation is very low (<1%).^40^, ^61^, ^62^ Pituitary adenomas are reported in five *SDHD* mutation carriers; in two of these, both macroprolactinomas, the presence of LoH was proven.^22^, ^23^

### 3.5 SDHAF2 mutations

The *SDHAF2* gene, like *SDHD*, is affected by maternal imprinting; therefore, only those carriers who inherit the mutation via the paternal line will develop the disease. Only a few cases of PGL/PCC associated with *SDHAF2* mutations have been described, and these account for <1% of all cases of PGL.^29^ Germline pathogenic variants in *SDHAF2* have been seen only in association with HNPGLs.^31^, ^63^, ^64^, ^65^, ^66^, ^67^ Kunst et al describe a large family of 16 patients, 11 with a HNPGL, primarily at carotid body and vagal locations. Within this family, the presence of multiple HNPGLs was common, and no cases of metastatic disease were found.^65^

## 4 CONSEQUENCES OF SDH DEFICIENCY OR LOSS

In *SDHx* germline mutation carriers affected by a second hit, SDH loss of function leads to the accumulation of succinate in the tumor cells,^68^, ^69^, ^70^, ^71^, ^72^ which is the hallmark of tumorigenesis of these tumors This accumulation inhibits several α‐ketoglutarate dioxygenases, which are involved in the induction of the pseudohypoxia pathway and in epigenetic DNA modifications. Moreover, SDH deficiency or loss may lead to overproduction of reactive oxygen species (ROS) and to a “rewiring” of the cell's metabolism.

### 4.1 Accumulation of succinate induces the pseudohypoxia pathway

Tumors harboring a *SDHx* mutation have a strong hypoxic signature. PGL/PCCs have historically been closely associated with hypoxia, because these highly vascularized tumors arise either in tissues known to be susceptible to low oxygen levels (adrenal medulla, organ of Zuckerkandl), or in cells known to serve as oxygen sensors (carotid body).

The major regulator of hypoxia response is the transcription factor HIF. HIF activity is regulated by TCA cycle metabolites. HIF is a heterodimer and consists of two subunits, one α subunit and one β subunit. There are three different α‐subunits: HIF1α, HIF2α and HIF3α, and two different β subunits: HIF1ß (aryl hydrocarbon receptor nuclear translocator [ARNT1]) and ARNT2. Whereas the β subunits are constitutively expressed, the active α subunits HIF1α and HIF2α are degraded in the presence of oxygen and therefore function as gatekeepers in response to low oxygen. Under normoxic conditions, HIFα is continuously synthesized, and propyl hydroxylase domain (PHD) marks it for degradation, involving the activity of the VHL ubiquitination complex (pVHL). The hydroxylation reaction performed by the PHD enzymes requires oxygen and α‐ketoglutarate as substrates, as well as iron and ascorbate as cofactors.^73^ Thus, during hypoxia PHD becomes inactive, and as a result HIFα escapes pVHL recognition and degradation. The unmodified HIFα molecule translocates to the nucleus, where it forms a transcriptionally active HIFα heterodimer with a stable HIFβ subunit. This active transcription factor induces a wide variety of target genes involved in cellular adaptation to hypoxia as in angiogenesis, energy metabolism, and cell survival.

In a SDH deficient condition, the excess of accumulated succinate is shuttled from the mitochondrial matrix to the cytoplasm, where it competes with α‐ketoglutarate in binding to PHD and inhibiting PHD. This consequently leads to the stabilization of HIFα even in the presence of oxygen, a condition known as pseudohypoxia.^3^, ^68^, ^74^, ^75^, ^76^

HIFα regulates the transcription of several genes known to be involved in tumorigenesis, angiogenesis, extracellular matrix elements and energy metabolism. HIF1α and HIF2α share the target genes vascular endothelial growth factor (VEGF), glucose transporters 1 and 3 (GLUT1 and GLUT3) and hexokinase 2. HIF1α stimulates the expression of various glycolytic enzymes and HIF2α stimulates the expression of platelet‐derived growth factor (PDGF) and erythropoietin (EPO).^9^ Pollard et al showed the overexpression of HIF1α in *SDHx* tumors compared to tumors with other germline mutations,^71^, ^77^ while others studies showed overexpression of HIF2α in *SDHx* related tumors compared to sporadic PGL/PCC.^78^, ^79^, ^80^ The role of HIF3α in relation to tumorigenesis remains to be elucidated, although, previous studies have indicated that HIF3α may suppress the expression of genes induced by HIF1α and HIF2α (for review see Reference 81).

Heat shock proteins (HSPs) are molecular chaperones that are important for protein assembly, folding and stability and play a central role in cell proliferation, survival and tumor progression. HSP90 is involved in the stability of HIF1α.^9^ HSP90 has been shown to be overexpressed in metastatic PGL/PCC compared with benign PGL/PCC.^82^ Inhibition of HSP90 leads to downregulation of HIF1α and is a potential target for therapy in metastatic PGL/PCC.^83^

### 4.2 Accumulation of succinate leads to a hypermethylator phenotype

**Figure 2:**
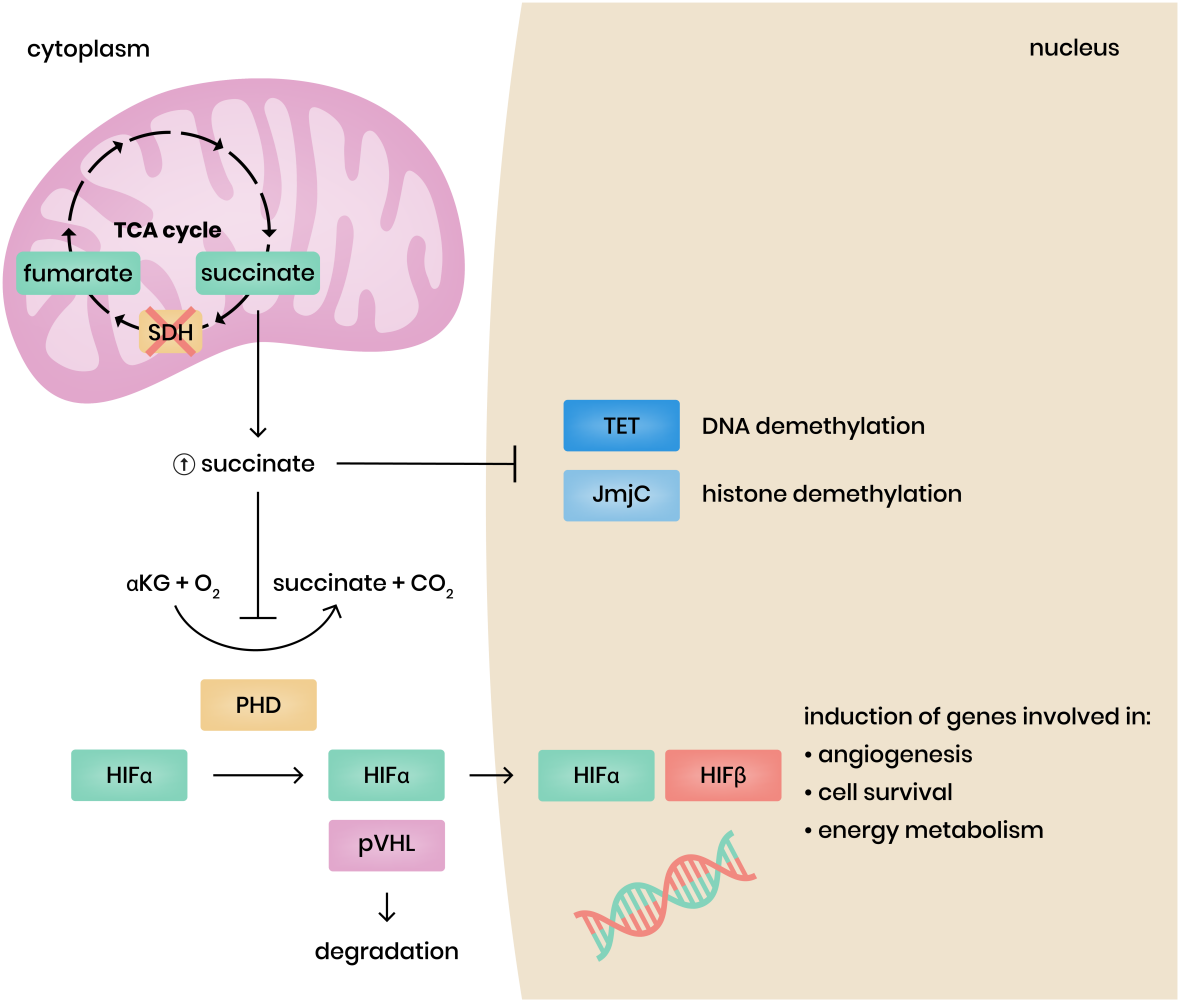
Consequences of succinate dehydrogenase (SDH) loss. SDH loss leads to the accumulation of succinate which inhibits a‐ketoglutarate dependent dioxygenases including prolyl‐hydroxylases (PHD), ten–eleven translocation (TET) and jumonji C‐domain‐containing proteins (JmjC) [Colour figure can be viewed at http://wileyonlinelibrary.com]

Recent studies have observed a hypermethylator phenotype in SDH deficient PGL/PCC.^84^, ^85^, ^86^ Next to PHD, accumulation of succinate competitively inhibits other α‐ketoglutarate‐dependent dioxygenases, including jumonji‐domain histone demethylases (JmjC) and the ten‐eleven translocation (TET) family of DNA methylase (Figure 2). Inhibition of these dioxygenases leads to hypermethylation of promotor regions (CpG islands) of several genes (also known as CpG island methylator phenotype [CIMP]). Because methylation triggers gene transcription deregulation, hypermethylation of tumor‐suppressor gene promotors plays an important role in tumorigenesis.

Letouzé et al determined the DNA methylation profiles of a large PGL/PCC cohort. They identified 191 genes showing significant hypermethylation, due to an inhibition of DNA demethylation, and downregulation in *SDHx*‐related PGL/PCC.^84^ The most significant epigenetically silenced genes were those encoding phenyl‐ethanolamine‐N‐methyltransferase *(PNMT)* and keratine 19 (*KRT19),* which are involved in neuroendocrine differentiation and in epithelial‐to‐mesenchymal transition (EMT).^84^

PNMT catalyzes the conversion of norepinephrine to epinephrine. Next to the *PNMT* gene, four other genes that we found to be hypermethylated, are involved in the catecholamine secretion: *SULT1A1*, *DRD2*, *NPY*, and *SLC6A2*.^84^ Reduced expression of these genes leads to an immature catecholamine secretory profile with predominant excretion of norepinephrine and dopamine. In *SDHB* mutated tumors the level of hypermethylation seems to be significantly higher compared to other *SDHx* mutated tumors, and the expression of these target genes significantly lower. The authors hypothesize that SDH inactivation may be more complete in *SDHB* mutated tumors compared to tumors harboring a mutation in the other subunits, leading to a higher succinate accumulation and hence a stronger inhibition of α‐ketoglutarate‐dependent demethylation.^84^ This could be an explanation for the higher metastatic risk in SDHB‐related tumors.

The study of Richter et al confirmed that tumor succinate:fumarate ratios were higher in tumors of patients with *SDHB* mutations compared to tumors of patients with an *SDHC/D* mutation.^87^ EMT is a process by which epithelial cells lose their polarity and cell‐to‐cell adhesion, thereby gaining migratory and invasive properties to become mesenchymal stem cells. This process, normally occurring during embryonic development, can be reactivated in cancer cells and is involved in metastatic dispersion.^88^ Several genes and signaling pathways have been identified as involved in different parts of the induction of EMT. *KRT19* encodes an intermediate filament required for the formation of desmosomes (structure specialized for cell‐to‐cell adhesion) and shown to be downregulated in *SDHB* metastatic PGL tissue samples unlike non‐*SDHB* metastatic PGL tissue samples.^89^ EMT is the first pathway identified that may be responsible for the specific metastatic properties of *SDHB*‐related PGL and PCC.

Kiss et al showed that the tumor suppressor gene *P16* was hypermethylated in *SDHB* mutated tumor tissue samples as opposed to *RET*‐, *VHL*‐ or *NF*‐related PGL/PCC.^90^ *P16* is an inhibitor of cyclin‐dependent kinases and plays an important role in cell cycle regulation by decelerating the cells progression from G1 phase to S phase, and acts therefore as a tumor suppressor. The authors showed that hypermethylation of *P16* was associated with short disease‐related survival.^90^

### 4.3 SDH loss leads to overproduction of reactive oxygen species

Reactive oxygen species (ROS) are damaging molecules containing oxygen with an unpaired free electron, such as superoxide and hydrogen peroxide. Although ROS are critical for normal cell function, they also lead to oxidative damage of DNA, which leading to genomic instability and finally to apoptosis. Mitochondria are the major source of ROS, especially complexes I and III, although complex II can also produce a significant number.^91^, ^92^ Accumulation of succinate results in an over‐reduced ubiquinone pool resulting in a reverse electron transfer to complex I, where electrons escape as ROS.^93^ Excessive ROS levels have been shown to stabilize HIFα and induce the pseudohypoxia pathway in *SDHx*‐mutated PGL/PCC.^94^ In addition to the stabilization of HIFα, *SDHx*‐mutation‐induced increases in ROS have been shown to cause genomic instability that may contribute to tumorigenesis.^95^, ^96^ Nevertheless, experimental evidence for ROS in various models of SDH dysfunction is not consistent, as some suggests that ROS are increased or normal, a finding which is extensively reviewed by Kluckova and coworkers.^8^

### 4.4 SDH loss leads to changes in the cell's metabolism pathways

**Figure 3:**
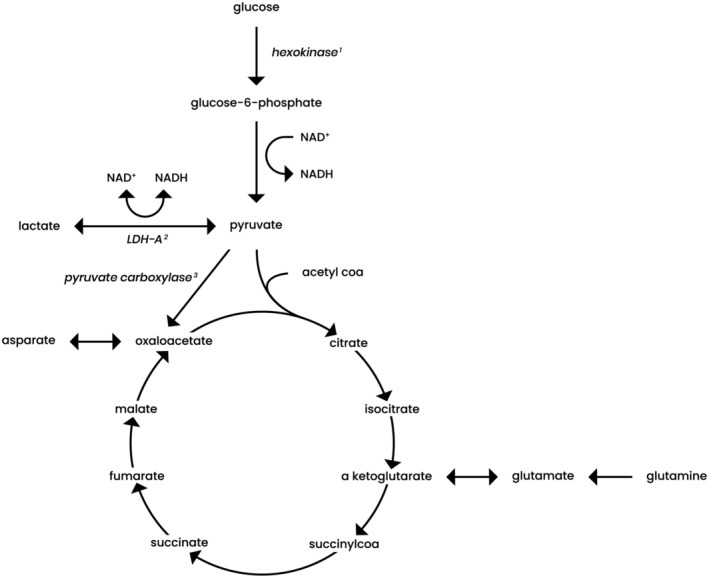
Metabolic pathways in which succinate dehydrogenase (SDH) loss is involved, including glycolysis, tricarboxylic acid cycle and anaplerotic reactions. The first step in glycolysis is the phosphorylation of glucose to glucose‐6‐phosphate by hexokinase^1^. Lactate dehydrogenase A (LDH‐A)^2^ catalyzes the conversion of pyruvate and lactate with concomitant conversion of nicotinamide adenine dinucleotide (NADH) and NAD^+^. Pyruvate carboxylase^3^ catalyzes the conversion of pyruvate to oxaloacetate. Proposed metabolic changes in SDH loss are enhanced glycolysis, via activation of LDH‐A and hexokinase. Furthermore, pyruvate carboxylase may be upregulated in SDH loss and there may be an increased glutamine metabolism. A more detailed explanation is described in the text

SDH deficiency or loss can lead to reprogramming of cancer‐related cell metabolism such as enhanced glycolysis (Warburg effect), as well as changes in anaplerotic pathways and in oxidative phosphorylation *(*Figure 3 *)*.

#### 4.4.1 Warburg effect

As stated above, *SDHx*‐related tumors display the Warburg effect. The main driver of the Warburg effect is HIFα which induces expression of GLUT1 and GLUT3, hexokinase 2, pyruvate kinase variant M2 (PKM2) and lactate dehydrogenase A (LDH‐A), thereby enhancing the glycolytic pathway.^97^ PKM2 interacts with HIF1α in the nucleus, where it functions as a coactivator to increase the expression of HIF1α target genes that stimulate the shift from oxidative phosphorylation to glycolysis.^98^, ^99^ Favier et al and Fliedner et al found an overexpression of LDH‐A in *SDHx*‐related tumors.^100^, ^101^ LDH‐A converts pyruvate to lactate, thereby recovering the NAD^+^ needed to maintain glycolysis, critical for tumor proliferation in vivo.^100^ The generated lactate leads to an acid tumor microenvironment, which in turn may facilitate tumor invasion and migration and is correlated with a poor prognosis.^102^

#### 4.4.2 Anaplerotic pathways

Pyruvate carboxylase, catalyzing pyruvate to oxaloacetate, an important anaplerotic reaction, may be upregulated in *SDHx* tumors. Cardaci et al showed that pyruvate carboxylase is upregulated in *SDHB* null cells. Silencing of the pyruvate carboxylase gene both significantly reduced the proliferation of SDH cells in vitro and delayed the onset of tumor in vivo, compared to SDH proficient cells/mice. By identifying pyruvate carboxylase as an essential gene for SDH‐deficient cells but dispensable for normal cells, this study unveils a metabolic vulnerability for potential treatment of *SDHx‐*associated tumors.^103^

Lussey‐Lepoutre et al showed that in *SDHx*‐mutated tumor cells the increased synthesis of oxaloacetate is essential in order to produce aspartate (as well as to continue a truncated oxidative TCA cycle). Aspartate is an important precursor for protein and nucleotide biosynthesis for anabolic purposes. In SDH deficient cells, as compared to wild type cells, knockdown of pyruvate carboxylase results in complete ablation of proliferation. The authors also showed the use of glutamine and glutamate to provide intermediates that are lacking due to TCA disturbance.^104^ Tannahill et al and Imperiale et al also showed an increased import and metabolism of glutamine in *SDHx‐*related tumors.^105^, ^106^

#### 4.4.3 Oxidative phosphorylation

Disruption of complex II leads to changes in the TCA cycle, but also to changes in oxidative phosphorylation in the form of upregulation of complex I. Pang et al showed that in tumor tissue and in an *SDHB‐* knockdown mouse cell line, complex I components and activity are upregulated.^107^ Consequently, the quantity of NAD^+^ in tumor tissue was 2.7‐fold higher in cluster I than in cluster II tumors. NAD^+^ is a cofactor that supports the poly (ADP‐ribose) polymerase (PARP) DNA repair way. PARP is an enzyme which produces ADP‐ribose‐conjugated PARP, involved in repair and stabilization of DNA. As an enhanced NAD^+^/PARP pathway was linked to chemoresistance in *SDHB* mutation carriers,^107^ inhibition of PARP could be a potential target to support chemotherapy, as further explained below.

## 5 APPLICATIONS FOR DIAGNOSTICS OF PGL/PCC

### 5.1 SDH and immunohistochemistry

In the vast majority of *SDHx*‐associated tumors, loss of SDHB protein expression can be detected by immunohistochemical staining with a high sensitivity and specificity (100% and 84%, respectively).^33^, ^108^ SDHB immunohistochemistry can therefore discriminate between *SDHx*‐related and non‐*SDHx*‐related PGL/PCC. Loss of both SDHB and SDHA immunoreactivity is shown only in the context of a *SDHA* mutation.^3^, ^23^, ^33^, ^61^, ^108^ SDHB and/or SDHA immunohistochemical expression could precedegenetic testing,^33^ or be used to classify variants of unknown significance.

The presence of an *SDHB* mutation is a predictor of metastasis in PGL/PCC. The current definition of a metastatic PGL or PCC according to the World Health Organization includes the presence of metastasis in non‐chromaffin tissue.^2^ In spite of attempts to develop an effective system for predicting the metastatic potential of PGL/PCC, none has yet resulted in a reliable classification. Recently, a grading system for PCC and PGL (GAPP) was developed.^109^ This score combines pathological features with biochemical phenotypes but does not include the *SDHB* mutational status of the tumor. Therefore, a combination of the GAPP and SDHB immunohistochemistry (modified‐GAPP or M‐GAPP) has been suggested as a valuable tool for predicting metastatic disease.^109^ Koh et al validated the M‐GAPP score in a retrospective cohort of 72 PGL/PCC patients with a mean follow‐up of 44 months. The M‐GAPP score was significantly higher in the 12 patients who developed metastatic disease.^110^

### 5.2 Metabolomics: measuring succinate levels in plasma, urine and tumor tissue

Succinate can be measured in plasma, urine and tumor tissue. Hobert et al measured succinate concentrations using gas chromatography‐mass spectrometry in plasma and urine of patients with germline *SDHB, SDHD, PTEN* mutations and patients with sporadic PGL/PCC. In three out of six *SDHx* mutation carriers (without PGL) elevated plasma succinate was recorded, while it was not elevated in any of patients with sporadic PGL/PCC.^111^

Tumor tissue can be used to measure the succinate:fumarate ratio using liquid chromatography‐mass spectrometry (LC‐MS).^112^, ^113^ An elevated succinate:fumarate ratio provides a diagnostic sensitivity of 93% and sensitivity of 97% to identify *SDHx* mutated PGL/PCC.^87^ Richter et al used 50 frozen specimens from 49 patients as a training set and 184 tumor samples as a validation set. In their study, succinate:fumarate ratios were higher in *SDHB*‐related PGL/PCC compared to *SDHC/D* tumors,^87^ thereby supporting Letouzé's suggestions^84^ that in a more complete inactivation of the SDH protein is present in *SDHB*‐mutation carriers. Measuring the succinate:fumarate ratio in tumor tissue can help to identify the underlying germline or somatic pathogenic mutation, especially when genetic mutation is inconclusive. Whether it may also have a prognostic value to predict metastatic disease still needs to be determined.

The studies of Lendvai et al and Imperiale et al confirm the findings that succinate:fumarate ratios are higher in *SDHB‐* and *SDHD*‐related PGL/PCC than in apparently sporadic and non‐*SDHx*‐mutated PGL/PCC (n = 8).^72^, ^106^ Imperiale et al also found significantly lower levels of glutamate in *SDHx*‐related tumors.^106^ In an additional study, Richter et al, used LC‐MS to screen 395 PGL/PCC tissues for TCA cycle metabolites to indicate TCA cycle aberrations. *SDHx‐*mutated tumors were characterized by high succinate levels and low levels of all other TCA cycle metabolites including glutamate and aspartate.^112^

High resolution magic angle spinning (HR‐MAS) nuclear magnetic resonance (NMR) spectroscopy is a new technique that can be used to analyze catecholamine and succinate levels both in vivo and ex vivo. The HR‐MAS NMR technique was used by the group of Taïeb to investigate the metabolic profile of *SDHx*‐mutated tumor tissue and to compare this profile to the metabolic profile of apparently sporadic and VHL tumor tissue.^114^ *SDHx*‐related tumors had increased levels of succinate and significantly decreased levels of glutamate compared to apparently sporadic tumors and *VHL*‐related tumors.^106^ The same group also explored the possibility of quantification of oncometabolites in tissue when the tumor is still inside the patient, and shown in eight patients that ^1^H‐MRS (^1^high magnetic resonance spectroscopy) adequately detected succinate resonance peaks in four patients with an *SDHx*‐related tumor.^115^ In addition, Lussey‐Lepoutre et al used ^1^H‐MRS to detect succinate levels in both mice and patients with PGL in vivo. Five patients had a *SDH*x gene mutation and in these patients a succinate peak could be detected.^116^ This offers unique opportunities for better characterizing these tumors at a metabolic level.

### 5.3 Altered cell metabolism pathways: The use of ^18^F‐fluorodeoxyglucose positron emission tomography

According to Endocrine Society PGL/PCC guidelines, ^18^F‐fluorodeoxyglucose (FDG) positron emission tomography (PET)/computed tomography (CT) is the preferred imaging modality in *SDHB‐*mutated PGL/PCC.^117^ Recent studies have shown that *SDHx*‐related PGL/PCC might be better visualized by [^68^Ga]‐DOTA(0)‐Tyr(3)‐octreotate ([67GA]‐DOTATATE) PET/CT than ^18^F‐FDG PET/CT, especially those located in the head and neck region as well as metastatic PGL/PCC.^118^, ^119^, ^120^ The sensitivity of FDG‐PET for *SDHx* related tumors varies between 83% and 100%.^118^, ^121^, ^122^, ^123^

Like glucose, ^18^F‐FDG is taken up by tumor cells mostly via GLUT. After cell entry, ^18^F‐FDG is phosphorylated by hexokinase into ^18^F‐FDG‐6‐P, which, unlike glucose‐6‐P, cannot be further metabolized along the glycolytic pathway. Because the cell membrane is impermeable to ^18^F‐FDG‐6‐P, it accumulates within cells in a manner directly proportionate to their metabolic activity. An increased glucose uptake and consumption due to an increase in glycolysis leads to a high uptake of ^18^F‐FDG.^124^ ^18^F‐FDG uptake in any cell is determined by expression of GLUTs and activity of hexokinase. Van Berkel et al studied the expression of GLUT and hexokinase in 27 tumor tissues from patients with hereditary tumors, using immunohistochemical staining and analyzed preoperative ^18^F‐FDG PET scans. The expression of hexokinase‐2 and hexokinase‐3 was significantly higher in *SDHx*‐mutated PGL/PCC than in sporadic tumors, and the mean standardized uptake values of the ^18^F‐FDG PET scans correlated with the expression of hexokinase‐2 and ‐3.^124^

Increased levels of succinate may also play a role in the high uptake of ^18^F‐FDG by *SHDx*‐related tumors. Garrigue et al showed that intratumoral injection of succinate significantly increased ^18^F‐FDG uptake in vivo and in vitro.^125^ Moreover, laser‐doppler did not show succinate induced ^18^F‐FDG uptake to be because of increased blood flow or increased capillary permeability.^125^

## 6 IMPLICATIONS FOR TREATMENT OF METASTATIC PGL/PCC

The cornerstone treatment for patients with benign *SDHx* related PGL/PCC is surgery.^117^ As described above, *SDHB*‐mutation carriers are those especially at risk of metastatic disease. Even for patients with metastatic PGL/PCC, resection of the primary tumor seems to be associated with a better overall survival.^126^ Metastases frequently occur in lymph nodes (distant and regional), bones, liver and lungs. Until now, there is no curative therapy for patients with metastatic disease. The main focus of treatment is on controlling the secretion of catecholamines, thereby alleviating symptoms and controlling tumor‐related complaints. Systemic treatment options include radionuclide therapy using ^131^I Metaiodobenzylguanidine (MIBG), peptide receptor radionuclide therapy (PRRT) and chemotherapy. As described above, insight in the pathways leading to tumor formation and potential metastatic disease in patients with *SDHx* mutations, may lead to a better response to existing therapies and provide us with a unique opportunity to develop novel targeted therapies.

### 6.1 Targeting the pseudohypoxia pathway

#### 6.1.1 Restoration of PHD activity

Succinate competes with α‐ketoglutarate in binding to PHD, thereby inhibiting PHD activity; therefore excess of α‐ketoglutarate could restore PHD.^11^, ^127^, ^128^, ^129^ Increasing levels of intracellular α‐ketoglutarate have been shown to affect the levels of HIF1α in vitro.^127^ As succinate and hypoxia act synergistically in inhibiting PHD activity, not only administering α‐ketoglutarate but also inducing hyperoxia might restore PHD activity.^130^, ^131^ Increasing the α‐ketoglutarate levels in the cell, is challenging. In a recent mouse model study of breast cancer, the α‐ketoglutarate dehydrogenase (KGDH) inhibitor (AA6) was able to cause intracellular α‐ketoglutarate accumulation.^132^

#### 6.1.2 HIF2α inhibition

In the HIF2α structure is a specific cavity which can be targeted.^133^, ^134^ Two compounds, PT2385 and PT2399, have been developed to serve as an HIF2α inhibitor. Both compounds, studied in vitro and in vivo, efficiently reduced the growth of clear cell RCCs.^135^, ^136^ A recent publication describes a phase I trial with PT2385 in patients with progressive clear cell RCCs. All 25 patients included in the expansion phase had locally advanced disease or disease that had progressed during a median of four prior regimens. Respectively, 2%, 12%, and 52% of patients had complete response, partial response and stable disease, results which are very promising.^137^ Although at present no intervention studies are being undertaken in patients with metastatic *SDHx‐* related PGL/PCC, probably in the near future a phase II trial will start to evaluate HIF2α inhibitors for patients with metastatic PGL/PCC.^133^, ^138^

#### 6.1.3 Tyrosine kinase inhibitors

Treatment with Tyrosine Kinase Inhibitors (TKI) targets the downstream pathway of HIF. Several TKI's that been described in case reports, series or phase II trials, such as sunitinib, cabozantinib, lenvatinib, pazopanib, and axitinib. An excellent overview of existing data and forthcoming trials was recently published by Toledo and Jiminez.^139^ All TKIs inhibit angiogenesis, by inhibiting the activation of the VEGF receptor (VEGFR).

Until now, most available data are for sunitinib. Besides inhibiting the activation of the VEGFR, sunitinib also inhibits the activation of the PDGF receptor and the receptor of RET, c‐KIT and Fms‐like tyrosine kinase (FLT). Canu et al reviewed the efficacy of sunitinib in 35 patients, of whom 13 were carriers of an *SDHB* germline mutation. Outcome did not differ between patients with or without an *SDHB* mutation.^140^ In a retrospective analysis of 17 patients with progressive disease, who received sunitinib, 47% had a partial response or stable disease. Positive responses were noted in carriers of *SDHB* mutations as well as in patients with apparently sporadic tumors. Progression‐free survival was only 4.1 months.^141^ Currently two phase II studies are being conducted; the Study Of Sunitinib In Patients With Recurrent Paraganglioma/Pheochromocytoma SNIPP (closed) and the First International Randomized Study in Malignant Progressive Pheochromocytoma and Paraganglioma (FIRSTMAPP).

Pazopanib, similar to sunitinib, also inhibits the action of VEGFR, PDGFR and the RET receptor, but additionally inhibits the fibroblast growth factor receptor (FGFR). Pazopanib was studied in a phase II trial, terminated due to poor accrual after including only seven patients.^142^ Six patients were evaluated, as one withdrew informed consent. Of the six only one patient had a partial response, lasting 2.4 years.

Preliminary results of axitinib were presented at the ASCO meeting in 2015. Axitinib only blocks VEGFR, and led to a partial response in three out of nine patients with metastatic PGL/PCC; moreover toxicity led to a high rate of dose reduction.^143^

Cabozantinib seems a promising TKI for patients with metastatic PGL/PCC, especially for patients with bone metastases. Cabozantinib also inhibits the c‐Met receptor pathways and may therefore delay the development of resistance, as this pathway is upregulated by VEGFR inhibition. Currently there is a phase II trial ongoing, with promising preliminary results.^144^

Another phase II trial is aiming to evaluate the response rate of lenvatinib in a group of 25 patients with metastatic PGL/PCC. Lenvatinib, like pazopanib, also inhibits FGFR.

#### 6.1.4 Heat shock protein 90 inhibitors

Inhibition of HSP90 leads to downregulation of HIF1α and is a potential target for therapy in metastatic PGL/PCC. Giubellino et al showed potent inhibition of proliferation in PCC cell lines by tanespimycin (17‐AAG) and ganetespib. Furthermore, they showed the efficacy of 17‐AAG and ganetespib in reducing metastatic burden and increasing survival in a metastatic model of PCC.^83^ Chae et al suggested that HSP90 could be especially effective in *SDHB*‐mutated tumors. Genetic inactivation of SDHB leads to a recruitment of HSP90 to the mitochondria, to help compensate for the impaired oxidative phosphorylation. As HSP90 promotes the stability of HIFα, its inhibition can lead to the death of these cells.^145^

### 6.2 Targeting the hypermethylator phenotype of *SDHx* related PGL/PCC

Chemotherapy is, in contrast to therapies mentioned above, widely available for the treatment of metastatic PGL/PCC. The combination of cyclophosphamide, vincristine and dacarbazine (CVD) is the most studied and is currently first line chemotherapy in patients with a metastatic PGL/PCC. However, in the absence of prospective studies, the evidence is only based on small retrospective studies.^141^, ^146^, ^147^, ^148^, ^149^, ^150^, ^151^ In 2014, a meta‐analysis was performed suggesting a partial response of 37%.^152^

Some reports however, suggest a higher response rate to temozolomide, an oral alternative to dacarbazine, in patients with *SDHB* mutations. Temozolomide is a DNA alkylating agent, leading to methylation of the O6‐position of guanine, resulting in DNA adduction. These DNA adducts result in apoptosis of the malignant cell. The O(6)‐methylguanine‐DNA‐methyltransferase (MGMT) enzyme is capable of repairing the DNA adducts. Therefore, the efficacy of treatment with temozolomide is associated with the expression of MGMT in the tumor cells. In a study by Hadoux et al, 11 out of 14 patients with progressive metastatic disease, had a *SDHB* mutation.^153^ Thirty‐six percent had partial response, 55% stable disease and 9% progressive disease. The authors observed a longer progression‐free survival in patients with an *SDHB* mutation compared to patients without an *SDHB* mutation (19.7 vs 2.9 months). The higher response rate in patients with *SDHB* mutations could be caused by hypermethylation of the MGMT promotor region and consequently lower MGMT expression.

Recently two patients with a *SDHB* metastatic PGL/PCC showed a clinical benefit from temozolomide even after disease progression on CVD. Both patients showed hypermethylation of the MGMT promotor region, suggesting that monotherapy of temozolomide may benefit patients with metastatic *SDHB‐*related PGL/PCC.^154^ Very recently Jawed et al studied 12 patients with a metastatic PGL/PCC, all with *SDHB* mutation, who received CVD; a marked efficacy was noted.^155^ Two out of 12 patients had a complete remission and eight patients a partial response. The median duration of response was 478 days, with a median progression‐free survival of 930 days.

Decitabine, registered for the treatment of acute myeloid leukemia, is a cytidine deoxynucleoside‐analog. It inhibits DNA‐methyltransferase and therefore acts as a hypomethylating agent. In two cell models decitabine was able to induce cell death of *SDH −/−* cells.^84^, ^156^

### 6.3 Preventing ROS damage

Ascorbic acid, α‐tocopherol (vitamin E) and N‐acetylcysteine all act as antioxidants preventing ROS damage, thereby diminishing tumorigenesis primarily through decreasing DNA damage and mutations. There is, however, limited evidence for this efficacy in *SDH*x mutated PGL/PCC.^157^, ^158^

### 6.4 Targeting the altered cell's metabolism

#### 6.4.1 Inhibiting glycolysis

As discussed above, *SDHx*‐related PGL/PCC are “glucose addicts.” Interventions aiming to inhibit glycolysis could therefore be an interesting and several potential options exists. WZB117 and STF‐31 are inhibitors of GLUT1, downregulating glycolysis and inhibiting cancer cell growth in vitro and vivo.^159^ Dichloroacetate (DCA) downregulates pyruvate dehydrogenase kinase. (Normally this upregulates pyruvate dehydrogenase involved in the glycolysis). This shifts glycolysis to oxidative phosphorylation and induces apoptosis in cancer cells. Pyruvate carboxylase was identified as an essential gene for SDH‐deficient cells (although dispensable for normal cells), a metabolic vulnerability offering a potential target for treatment of *SDHx‐*associated tumors.^103^

#### 6.4.2 Inhibiting the effects of upregulation of complex I

As noted above another way to become resistant to chemotherapy is via the NAD^+^/PARP‐pathway. In the study of Pang et al the combination of temozolomide with a PARP inhibitor led to increased mouse survival in a metastatic PGL/PCC allograft model (52 days compared with 42 days).^107^ Notably, one of the postulated pathways through which metformin exerts an anti‐tumor effect is also through inhibition of complex I, implying that metformin could also act as a potential chemosensitizer in patients with *SDHx*‐related metastatic PGL/PCC.

## 7 CONCLUSION

Recent years, we have seen an increase in knowledge regarding the consequence of loss of the SDH enzyme in the pathogenesis of (metastatic) PGL/PCC in patients harboring an *SDHx* mutation. The accumulation of succinate and the impairment of the complex II function of oxidative phosphorylation leads, via the pseudohypoxic pathway, induction of ROS, and rewiring of the cell's metabolism to tumor formation. The advantages of new insight into these pathophysiological characteristics provide new directions for diagnostics and therapeutic options in metastatic *SDHx*‐related PGL and PCC.

## CONFLICT OF INTEREST

All authors hereby declare that they are no conflicts of interest. Data sharing is not applicable to this article as no new data were created or analyzed in this study.
